# Protective Effect of the Silkworm Protein 30Kc6 on Human Vascular Endothelial Cells Damaged by Oxidized Low Density Lipoprotein (Ox-LDL)

**DOI:** 10.1371/journal.pone.0068746

**Published:** 2013-06-28

**Authors:** Wei Yu, Huihui Ying, Fudan Tong, Chen Zhang, Yanping Quan, Yaozhou Zhang

**Affiliations:** 1 Institute of Biochemistry, College of life sciences, Zhejiang Sci-Tech University, Zhejiang Province, Hangzhou, China; 2 Zhejiang Provincial Key Laboratory of Silkworm Bioreactor and Biomedicine, Zhejiang Province, Hangzhou, China; William Harvey Research Institute, Barts and The London School of Medicine and Dentistry, Queen Mary University of London, United Kingdom

## Abstract

Although the 30K family proteins are important anti-apoptotic molecules in silkworm hemolymph, the underlying mechanism remains to be investigated. This is especially the case in human vascular endothelial cells (HUVECs). In this study, a 30K protein, 30Kc6, was successfully expressed and purified using the Bac-to-Bac baculovirus expression system in silkworm cells. Furthermore, the 30Kc6 expressed in *Escherichia coli* was used to generate a polyclonal antibody. Western blot analysis revealed that the antibody could react specifically with the purified 30Kc6 expressed in silkworm cells. The *In vitro* cell apoptosis model of HUVEC that was induced by oxidized low density lipoprotein (Ox-LDL) and *in vivo* atherosclerosis rabbit model were constructed and were employed to analyze the protective effects of the silkworm protein 30Kc6 on these models. The results demonstrated that the silkworm protein 30Kc6 significantly enhanced the cell viability in HUVEC cells treated with Ox-LDL, decreased the degree of DNA fragmentation and markedly reduced the level of 8-isoprostane. This could be indicative of the silkworm protein 30Kc6 antagonizing the Ox-LDL-induced cell apoptosis by inhibiting the intracellular reactive oxygen species (ROS) generation. Furthermore, Ox-LDL activated the cell mitogen activated protein kinases (MAPK), especially JNK and p38. As demonstrated with Western analysis, 30Kc6 inhibited Ox-LDL-induced cell apoptosis in HUVEC cells by preventing the MAPK signaling pathways. *In vivo* data have demonstrated that oral feeding of the silkworm protein 30Kc6 dramatically improved the conditions of the atherosclerotic rabbits by decreasing serum levels of total triglyceride (TG), high density lipoprotein cholesterol (HDL-C), low density lipoprotein cholesterol (LDL-C) and total cholesterol (TC). Furthermore, 30Kc6 alleviated the extent of lesions in aorta and liver in the atherosclerotic rabbits. These data are not only helpful in understanding the anti-apoptotic mechanism of the 30K family proteins, but also provide important information on prevention and treatment of human cardiovascular diseases.

## Introduction

The 30K family proteins are low molecular weight lipoproteins (LP) that are expressed in hemolymph of the silkworm larvae. It has been confirmed that the silkworm protein 30Kc6 is one of the members of the 30K family proteins that transports lipids and inhibits cell apoptosis in the insect and mammalian cells [1]–[5]. However, effects of 30Kc6 on cell apoptosis of human vascular endothelial cell (HUVEC) and the underlying mechanism are largely unknown.

Atherosclerosis (AS) is a vascular system disease with characteristics of non-inflammatory state, retrogression and hyperplastic pathologies. It often occurs in carotid arteries, aortas and peripheral arteries and seriously threatens human health [6]–[9]. Vascular endothelial cell (VEC) is a blood-brain barrier and a common target of Ox-LDL, angiotensin II (Ang II), high glucose and other risk factors [6]. Furthermore, VEC apoptosis plays a critical role in the pathogenesis of AS. It has been confirmed that apoptosis of VEC was an important initiating step for AS and was further involved in the whole process. Moreover, the VEC apoptosis played a key role in induction of atherosclerotic lesion formation and plaque shedding. Therefore, prevention of the oxidative stress-induced HUVEC damage might be one of the methods in the prevention and treatment of AS [7]–[9].

Mitogen activated protein kinases (MAPK), serine/threonine kinases in most cells, are important molecules that accept and transmit the receptor-mediated extracellular signaling into cytoplasm and nucleus in order to participate in the gene expression and regulation as well as cell proliferation and cell death especially in eukaryotes. Extracellular receptor-activated kinases (ERK), c-Jun N-terminal kinases (JNK) and p38 mitogen-activated protein kinase (p38 MAPK) are three major signaling kinases that are involved in cell apoptosis [10]. Specifically, JNK and p38 are oxidative stress-induced MAPK and are activated by intracellular oxidative stress that leads to cell apoptosis [11]–[12].

Therefore, in this study, the silkworm protein 30Kc6 was expressed and purified using the Bac-to-Bac Baculovirus expression system. The effects of 30Kc6 on Ox-LDL-induced VEC apoptosis and apoptotic signaling pathways were then investigated in HUVEC cells. In addition, the protective effects of the silkworm oral feeding with pupa meal containing the 30Kc6 protein were further analyzed in atherosclerotic rabbit animal models.

## Materials and Methods

### Materials

The cultured silkworm BmN cells, the recombinant prokaryotic expression vector pET-28a-*30Kc6*, plasmid pFastBac-HTB, virus vector Bacmid, *Escherichia coli* BL21 (DE3) and DH10Bac stains were propagated or constructed in our laboratory. HUVEC cells were purchased from ATCC (Manassas, VA, USA). New Zealand white rabbits were purchased from Experimental Animal Center of Hangzhou Normal University (China, SCXK (Zhe) 2010-0047). For experiments involving animals, approval was obtained from the Institutional Review Committee of Hangzhou Normal University. High fat diets were purchased from Xietong Medical Biological Engineering Limited Liability Company (Jiangsu, China). The cell culture medium sf-900 for insect cells, mammalian cell culture medium DMEM, Hanks buffer and fetal bovine serum (FBS), complete and incomplete Freund’s adjuvant were purchased from Gibco (Grand Island, NY, USA). Ni-NTA Purification System was sourced from Invitrogen (Carlsbad, CA, USA). LDL was purchased from Calbiochem-Merck (KGaA, Darmstadt, Germany). The Mouse 6×His-tag monoclonal antibody, Cell Death Detection ELISA kit and Cell Proliferation ELISA kit were purchased from Roche (Mannheim, Germany). Horseradish peroxidase (HRP)-conjuagated goat-anti-mouse-IgG antibody and goat-anti-rabbit-IgG antibody were purchased from Beijing Dingguo Biotechnology Company (Beijing, China). The 8-isoprostane EIA kit was purchased from Cayman chemical (Ann Arbor, MI, USA). Monoclonal antibodies anti-phospho-p38 MAP kinase (phospho-p38), anti-p38 MAP kinase, anti-JNK MAP kinase (phospho-JNK) and anti-JNK MAP kinase were purchased from Cell Signaling Technology Company (Beverly, MA, USA). The protein A sepharose CL-4B column and enhanced chemiluminescence (ECL) detection system were purchased from Amersham Pharmacia Biotech (Piscataway, NY, USA). The Malondialdehyde (MDA) detection kit, kits of total cholesterol (TC), total triglyceride (TG), LDL and high density lipoprotein (HDL) and oil red O stain were purchased from Jiancheng Science and Technology Limited Company (Nanjing, China). Other reagents were purchased from Sigma (St. Louis, MO, USA).

### Construction of Recombinant Virus Vector Bacmid-30Kc6

According to the *30Kc6* gene sequence (GenBank No. X54735), the PCR primers were designed to amplify the *30Kc6* gene. The promers used include the forward primier, 30Kc6F: 5′-CGCGGATCCATGAGACTGACTTTGTTT-3′ and the reverse primer, 30Kc6R: 5′- CCGCTCGAGTTAGTAGGGGACGATGTA-3′. The *30Kc6* gene was inserted into the MCS of the transfer plasmid pFastBac-HTB between *Bam*H I and *Xho* I sites, and it was transformed into the DH10Bac cells. The *30Kc6* gene was then transferred into Bacmid DNA by homologous recombination to construct the recombinant baculovirus Bacmid-30Kc6. After white-blue plaque selection, the positive colonies were selected and analyzed by PCR with M13 universal primers and *30Kc6* forward and reverse primers. The recombinant virus was further confirmed by DNA sequence analysis.

### Expression and Purification of the Silkworm Protein 30Kc6

The BmN cells in logarithmic growth phase were infected with recombinant virus Bacmid-30Kc6 with a multiplicity of infection (MOI) of 10. The infected BmN cells with obvious infection symptoms were harvested in 72 hours (h) and were centrifuged at 1000 rpm for 10 min. The harvested cells were washed with phosphate buffered saline (PBS) and were centrifuged at 1,000 rpm for 10 min. The cells were suspended in 200 µL PBS and were lysed by ultrasound on ice. Cell lysates were centrifuged for 20 min with a speed of 12,000 rpm and the supernatants were harvested. The 1× native binding buffer (pH 8.0) was used to balance nickel column and the cell lysates were loaded into the nickel column and were incubated overnight on ice. The native washing buffer (pH 8.0) with a concentration gradient of imidazole was employed to wash the columns in batches. Finally, the silkworm protein 30Kc6 was purified by eluting the binding proteins with the native elution buffer (pH 8.0).

### Preparation of Polyclonal Antibodies

*Escherichia coli* BL21 (DE3) was transformed with the the recombinant prokaryotic expression vector pET-28a-*30Kc6* and was cultured in LB media with 50 µg/mL kanamycin, at 37°C until the OD_600_ reached approximately 0.5. Recombinant protein expression was induced with 1 mmol/L IPTG for 4 h. The His-tagged fusion protein was extracted from the bacteria and purified by Ni^2+^-affinity chromatography. The purified protein was subsequently concentrated and desalted by dialysis. After thrombin digestion, 30Kc6 was released from the fusion protein. Then the protein was analyzed following the method of Bradford. A primary immunization of 0.5 mg/rabbit of 30Kc6 protein with Complete Freund’s Adjuvant (1∶1) was administered to male New Zealand white rabbits on day 0. Boost immunizations of 0.5 mg 30Kc6 protein with Incomplete Freund’s Adjuvant were administered on days 21. After boosted 3 times with 30Kc6 protein, the blood were collected and centrifuged at 4°C 10, 000 g for 10 minutes, pipetted off the supernatant to obtain the antiserum, stored at −20°C. Columns containing 10 ml of packed protein A sepharose CL-4B were used to purify the polyclonal antibody. An ELISA was utilized to determine the polyclonal antibody titer and the specificity of the polyclonal antibody was detected by Western blot analysis with purified 30Kc6 protein.

### Induction of Apoptosis in HUVEC

LDL was placed into the dialysis bag with proper diameter and was incubated with PBS for 24 h at 4°C. The CuSO_4_ was added into the PBS solution containing LDL with a final concentration of l0 µmol/L. The LDL was further incubated with CuSO_4_ for 24 h at 4°C. The oxidation-reduction reaction was stopped by putting the mixture into the PBS containing l µmol/L EDTA for 24 h at 4°C. Finally, the Ox-LDL was produced and sterilized by filtration with filter membrane (0.22 µm). The protein concentration of the prepared Ox-LDL was measured by the Bradford method. The malondialdehyde (MDA) value of Ox-LDL was 12 times of that of the LDL in MDA measurement analysis, indicating that the LDL was oxidated and could be stored at 4°C.

The HUVEC cells were used while they were in the logarithmic growth phase. They were plated in the 6-well microtiter plates at a density of 1×10^5^ cells/well and were cultured at 37°C overnight under 5% CO_2_. The culture media was discarded and the cells were washed twice with Hanks solution. They were then incubated with Ox-LDL with concentrations of 20, 50, 100 and 150 µg/mL for 24 h, respectively. The treated cells were washed three times with Hanks solution and cultured with cell complete media (10% FBS) for 24 h at 37°C with 5% CO_2_. Finally, the cell viability was measured with the Cell Proliferation ELISA kit (Roche) and cell apoptosis was determined with the Cell Death Detection ELISA kit (Roche) according to the directions of the kit.

### Evaluation of HUVEC Viability

The HUVEC cells in the logarithmic growth phase were plated in 96-well microtiter plates at a density of 2×10^3^ cells/well and were cultured at 37°C overnight under 5% CO_2_. The cultured cells were incubated with the purified silkworm protein 30Kc6 with a final concentration of 5 µg/ml for 24 h. The pre-treated cells were washed with Hanks solution twice and were further incubated with 100 µg/mL Ox-LDL for 24 h. The cells were washed three times with Hanks solution and were further treated with the purified silkworm protein 30Kc6 with a final concentration of 5 µg/ml for 24 h. Finally, the cell viability was measured with the Cell Proliferation ELISA kit (Roche) as described previously. The HUVEC cells without any treatment were set as the untreated blank control group. The HUVEC cells treated with 30Kc6 proteins were set as the 30Kc6 control group. The HUVEC cells incubated with Ox-LDL were set as the Ox-LDL control group and those cells incubated with both 30Kc6 and Ox-LDL were set as the experimental group 30Kc6+Ox-LDL. Every group was set with three duplicates.

### Evaluation of Apoptosis and 8-isoprostane Level in HUVEC Cells

The HUVEC cells in the logarithmic growth phase were used and plated in 96-well microtiter plates at a density of 2×10^3^ cells/well and were cultured overnight at 37°C under 5% CO_2_. The cells were treated with 30Kc6 or/and Ox-LDL as described previously. The treated cells were harvested, lysed and subjected to DNA fragmentation analysis with the Cell Death Detection ELISA kit (Roche).

To further examine the effects of 30Kc6 on the intracellular state of oxidative stress in Ox-LDL-induced HUVEC, the HUVEC cells in the logarithmic growth phase were used. They were plated in 35 mm culture dishes at a density of 5×10^4^ cells/dish and were cultured overnight at 37°C under 5% CO_2_. The pre-treated cells were harvested and lysed. The 8-isoprostane standard substances and the cell lysates were prepared according to the directions of the 8-isoprostane EIA kit (Cayman chemical). The absorbance of each well was measured at a wavelength of 405 nm by an Enzyme-Linked Immunosorbnent Assay (ELISA) reader.

### Immunoblotting

The HUVEC cells in the logarithmic growth phase were used and plated in 60 mm dishes at a density of 2×10^5^ cells/well and were cultured overnight at 37°C under 5% CO_2_. The cells were treated, harvested and lysed as described previously. Briefly, the cells were washed twice with ice-cold PBS and lysed in lysis buffer (25 mmol/l Tris/HCl with pH 7.5, 25 mmol/l NaCl, 0.5 mmol/l EGTA, 10 mmol/l NaF, 20 mmol/l h-glycerophosphate, 1 mmol/l Na_3_VO_4_, 1 mmol/l PMSF and 10 µg/ml aprotinin) at 4°C. After sonication and centrifugation at 15,000 rpm, the supernatant was used for immunoblotting. The lysate (20 µg protein per lane) was separated on 12% SDS-polyacrylamide gel, electroblotted onto nitrocellulose membrane and immunoblotted with specific primary antibodies. The antibodies used in this study were anti-phospho-p38 MAP kinase, anti-p38 MAP kinase, anti-phospho-JNK MAP kinase and anti-JNK MAP kinase (Cell Signaling Technology). The antibodies were detected by a horseradish peroxidase-linked secondary antibody using an enhanced chemiluminescence system (Amersham Pharmacia Biotech). Densitometric analysis was performed using an image scanner and analyzing software (NIH image ver. 1.61). The activity of each kinase was evaluated by calculating the ratio of the amount of the phosphorylated form to that of the total form.

### Determination of the Content of 30Kc6 Protein in Pupa Powder

Silkworm pupas were infected with Bacmid or the recombinant virus Bacmid-30Kc6. When silkworm pupas demonstrated obvious symptoms related to the virus infection in five days, they were freeze-dried and pupa powders were produced. The silkworm freeze-dried pupa powder that was infected with Bacmid-30Kc6 (1 g) and Bacmid (1 g) was diluted 100 times in PBS. A solution containing 5 µg/mL of previously purified 30Kc6 protein expressed in BmN cells was prepared, and a serial dilution of 30Kc6 was generated. The standard proteins were coated onto the 96-wells with the vehicle as controls. The coated wells were incubated with proteins for two hours at 37°C, followed by further incubation for more than 18 h at 4°C. The treated wells were washed with 0.02 mol/L PBS containing 1% Tween 20 (pH 7.4) and blocked with PBS containing 1% BSA for 2 h at 37°C. After washing once with PBS, the wells were incubated with the primary antibody (home-made polyclonal antibodies, 100 µL). The wells were washed three times with PBS and incubated with the horseradish peroxidase (HRP)-labeled goat-anti-rabbit secondary antibody (100 µL) for 1h. After washing three times, the wells were incubated with freshly prepared substrate solution (100 µL) for 10–15 min at 37°C and the reactions were stopped. The absorbance of each well was measured at a wavelength of 492 nm by an ELISA reader. The standard curve was obtained according to the standard substance, and the targeted protein content of silkworm pupa powder was calculated.

### Construction of Atherosclerotic Rabbit Models

Thirty New Zealand white rabbits were randomly divided into two groups including the normal control group (n = 5) fed with normal diet and the high-fat group (n = 25). The high-fat group was first given a bovine serum albumin (BSA) injection (250 mg/kg) from auricular vein at the beginning of this experiment and were then fed with high-fat diet (79% basic diet, 1% cholesterol, 10% lard and 10% egg yolk powder). Each rabbit was given 150 g food per day and had free access to water. All rabbits were housed in animal room for eight weeks and the blood concentration of total cholesterol (TC), triglyceride (TG), high-density lipoprotein cholesterol (HDL-C) and low-density lipoprotein cholesterol (LDL-C) were measured by kits according to the manufacturer’s directions. Eight weeks later, three rabbits in high-fat diet group were randomly selected. After euthanasia of the animals by intravenous injection of pentobarbital, the aorta and liver of each animal were obtained in order to assess the success of the atherosclerotic rabbit model construction by paraffin section examinations, as described previously [13].

### The Effects of 30Kc6 Protein on Atherosclerotic Rabbit

The normal diet group served as blank control. In contrast, rabbits in high fat diet group, which were successfully constructed atherosclerotic models, were divided into five different groups including: (1) high-fat group: animals were fed with normal diet; (2) Bacmid group: animals were fed with normal diet and an addition of Bacmid-infected freeze-dried silkworm pupa meal (20 mg/kg.d) through intragastric administration and normal diet; (3) high dose group: animals were fed with normal diet and an addition of Bacmid-30Kc6-infected silkworm pupa meal (30Kc6 20 mg/kg.d); (4) low dose group: animals were fed with normal diet and an addition of Bacmid-30Kc6-infected silkworm pupa meal (30Kc6 4 mg/kg.d); (5) positive control group: animals were fed with normal diet and addition of probucol (37.5 mg/kg.d). All groups were fed for four weeks.

Before the rabbits were sacrificed, the blood serum concentrations of 30Kc6 were examined in the rabbits. The blood (2 ml) was collected from the brachial vein with a single-use syringe after drug administration at 2 h. The blood samples were drawn into EDTA-coated anticoagulation tubes, mixed thoroughly and centrifuged at 3000 rpm. The serum samples were collected and stored at 4°C for use. The 30Kc6 concentrations were determined by ELISA using the home-made polyclonal antibody. The purified 30Kc6 expressed in BmN cells was used as the standard sample. The standard curve was generated as previously discussed. The 30Kc6 concentrations of the tested samples were calculated. Following the animal euthanasia by intravenous injection of pentobarbital, all rabbits in different groups were sacrificed by gas embolization after their bloods were drawn from their hearts. The aorta and liver of each animal were obtained in order to determine the lesions.

### Morphological Examination of Aortas and Livers

Haematoxylin and Eosin Staining: The abdominal aortas and livers from the rabbits were immediately dissected, fixed in 10% neutral buffered formalin, dehydrated and embedded in paraffin. The aortas were cut into 10 serial 2.5 mm sections. The tissue sections (10 µm thick) were cut from the paraffin-embedded blocks on a microtome and mounted from warm water (40°C) onto adhesive microscope slides. Sections were allowed to dry overnight at room temperature and were stained with hematoxylin-eosine (HE), as described previously [14].

Oil Red O Staining: The aortas were cut into 10 serial 2.5 mm sections and immediately frozen in liquid nitrogen. The frozen tissue blocks were placed on a cryotome, and 10 µm serial sections of the ascending aorta were collected on coated glass slides. Every fifth section of the ascending aorta was stained with oil red O stains.

The areas of the intima and of the media were measured by image analyzing software (NIH Image ver. 1.61) and the ratio of the intimal area to the medial area (I/M ratio) was calculated. The average of five sections was taken as the value for each animal [15]. Finally, the samples were examined for bubble lesions of livers under the light microscope.

### Statistical Analysis

Data described in this study were demonstrated as mean±SD (n = 3). Statistical significance between the means was determined and analyzed by one-way analysis of variance (ANOVA) and Student’s *t*–test using SPSS version 11.0 software. A *P* value of less than 0.05 was considered statistically significant and a *P* value of less than 0.01 was considered highly significant.

## Results

### Identification of Recombinant Virus Bacmid-30Kc6

**Figure 1:**
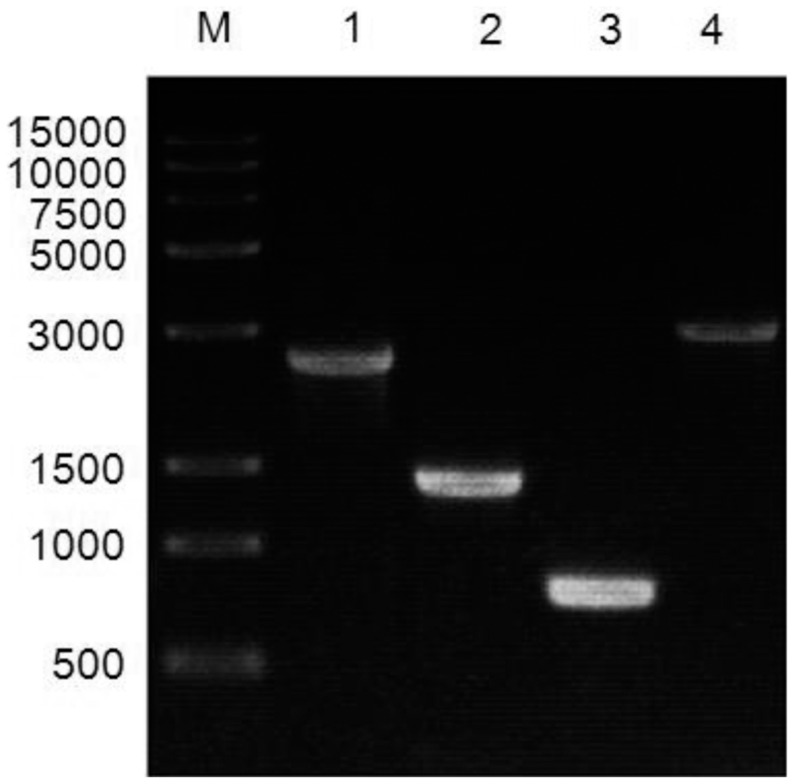
Polymerase chain reaction (PCR) confirmation of recombinant baculovirus Bacmid-30Kc6. M: DNA Marker; 1. PCR products after the use of M13F and 30Kc6R primers; 2. PCR products after the use of 30Kc6F and M13R primers; 3. PCR products after the use of 30Kc6F and 30Kc6R primers; 4. PCR products after the use of M13F and M13R primers.

After white-blue plaque selection, three single positive colonies were selected and were cultured in liquid LB media with chloromycetin and kanamycin overnight. The Bacmid DNA was extracted using the alkaline lysis method for PCR confirmation. With the PCR using Bacmid-30Kc6 DNA as template and M13F and 30Kc6R as primers, the PCR product was supposed to be 2421 bp long. With the 30Kc6F and M13R primers, the PCR product was supposed to be 1374 bp long. With the 30Kc6F and 30Kc6R primers, the PCR product was supposed to be 771 bp long. With the M13F and M13R primers, the PCR product was supposed to be 3111 bp long. Figure 1 showed that the length of the PCR products in each group were consistent with theoretical values, indicating that the recombinant virus Bacmid-30Kc6 was successfully constructed. The DNA sequence analysis further confirmed that *30Kc6* gene was successfully inserted into Bacmid genome (data not shown).

### Purification and Characterization of a Polyclonal Antibody to 30Kc6 Protein

**Figure 2:**
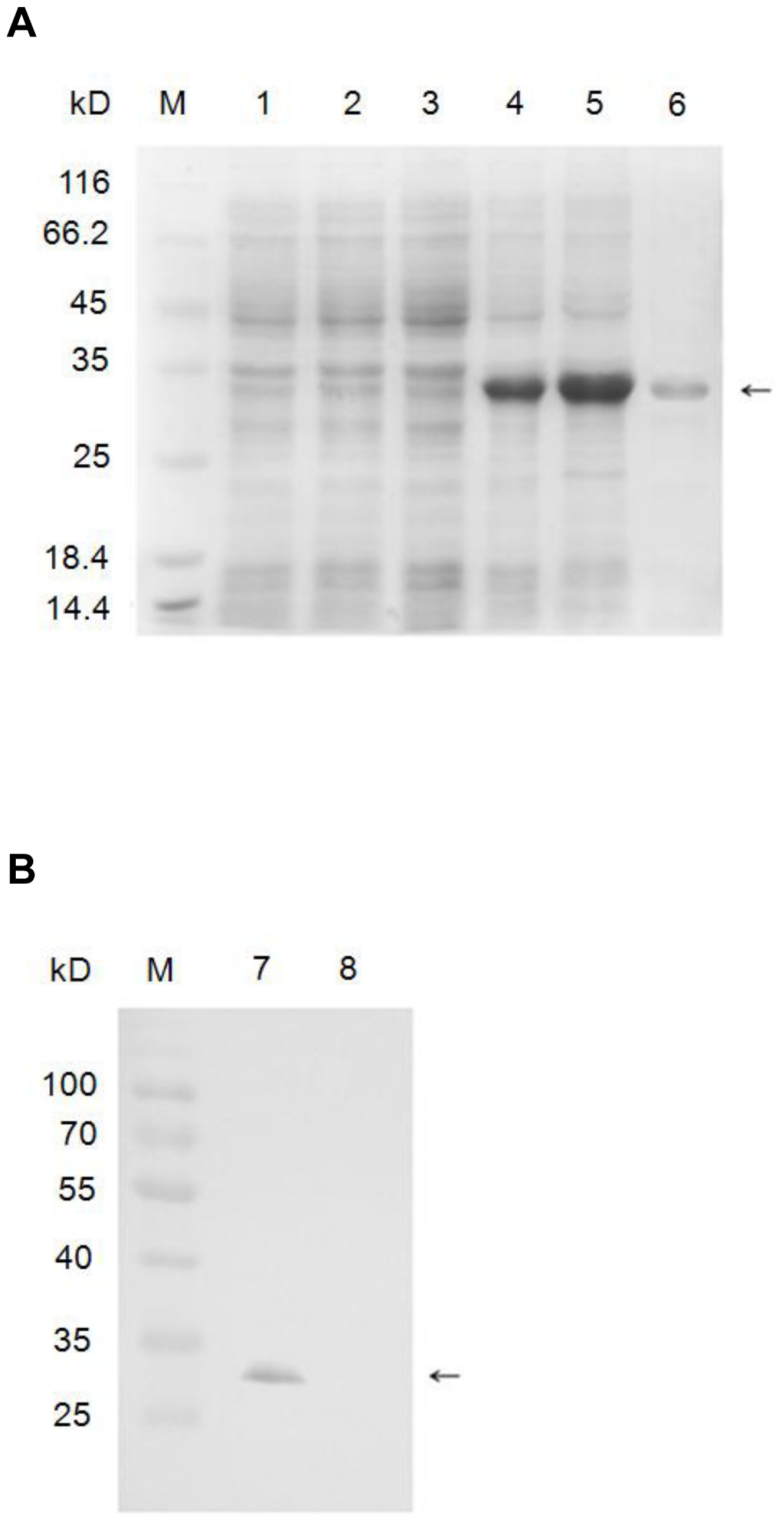
Expression, purification (A) and polycnonal antibody generation (B) of 30Kc6 protein in *E.* *coli*. The *30Kc6* gene was inserted into prokaryotic expression plasmid pET-28a and was expressed in *E. coli* BL21 (DE3) with the induction of IPTG. M. represents the protein molecular weight marker. 1. Empty vector before inducing; 2. Empty vector after inducing; 3. Recombinant bacteria before inducing; 4. Recombinant bacteria after inducing (IPTG 0.1 mmol/L); 5. Recombinant bacteria after inducing (IPTG 1 mmol/L); 6. Purified 30Kc6 protein; 7. Purified antibody reacts with purified 30Kc6 protein; 8. Pre-immune rabbit serum reacts with purified 30Kc6 protein. The arrows indicate the 30Kc6 protein.

A recombinant prokaryotic expression plasmid pET-28a-*30Kc6* was transformed into *Escherichia coli* BL21 (DE3) and induced with IPTG. The His-tagged fusion protein was purified by Ni^2+^-affinity chromatography (Fig. 2A). A 30Kc6 polyclonal antibody was generated by immunizing New Zealand white rabbits with the 30Kc6 protein purified as described in Materials and Methods. The specificity of the purified antiserum was confirmed by Western blotting. On immunoblots, the purified antibody specifically recognized the purified 30Kc6 protein expressed in *E. coli* and the band was of the expected about 30 kD molecular size. No bands were evident when the same sample was subjected to immunolblotting with the pre-immune rabbit serum (Fig. 2B). The titer of the polyclonal antibody against the 30Kc6 protein was about 1∶12800 as determine by indirect- ELISA.

### Purification and Identification of the Silkworm Protein 30Kc6 Expressed in BmN Cells

**Figure 3:**
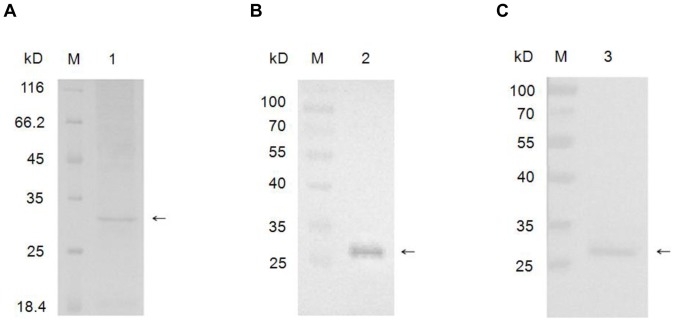
Analysis of the purified 30Kc6 expressed in BmN cells by SDS-PAGE (A) and Western blot (B, C). Purified 30Kc6 proteins were transferred to nitrocellulose membrane after SDS-PAGE and analyzed by immuno-blotting with anti-6×His-tag antibody and home-made polyclonal anti-30Kc6 antibody, respectively. M represents the protein molecular weight marker. 1. The purified silkworm fusion protein 30Kc6 in SDS-PAGE analysis; 2. The purified silkworm fusion protein 30Kc6 in Western blotting using the monoclonal anti-6×His-tag antibody; 3. The purified silkworm fusion protein 30Kc6 in Western blotting using the home-made polyclonal anti-30Kc6 antibody. The arrows indicate the 30Kc6 protein.

In order to investigate whether the silkworm protein 30Kc6 had any protective effect on HUVEC cells, which were incubated with Ox-LDL, silkworm protein 30Kc6 was expressed in BmN cells and was purified by Invitrogen Ni-NTA. It was then subjected to SDS-PAGE and Western blot analysis. As demonstrated in Fig. 3, there was an obvious 30KD fusion protein band corresponding to the molecular weight of the purified 30Kc6 protein (Fig. 3A). Furthermore, the purified protein showed excellent reaction specificity with 6×His-tag monoclonal antibody (Fig. 3B) and the home-make polyclonal antibody (Fig. 3C).

### HUVEC Cell Apoptosis Induced by Ox-LDL

**Figure 4:**
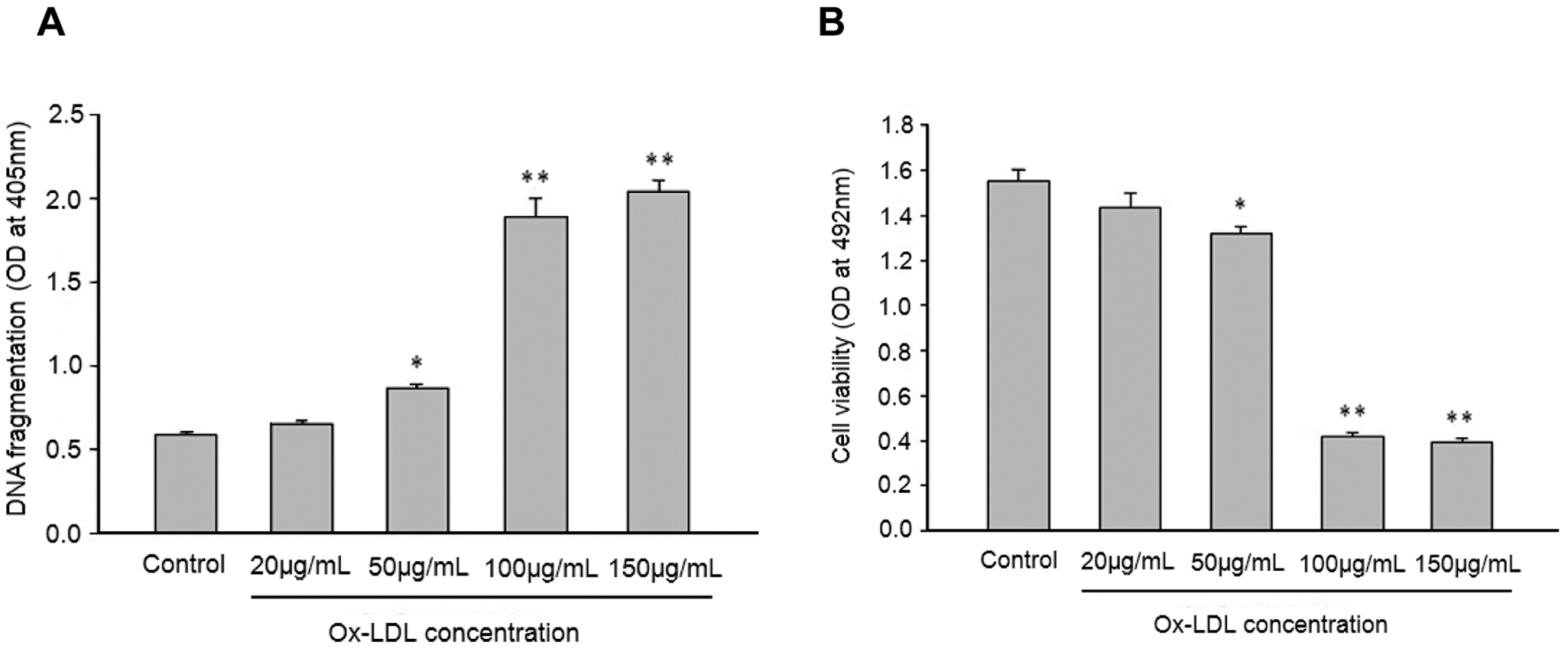
Results of the DNA fragmentation (A) and the cell viability (B) in HUVEC cells treated with Ox-LDL. The HUVEC cells were treated with different concentrations of Ox-LDL (20, 50, 100 and 150 µg/mL) for 24 h. The cell viability and apoptosis were analyzed. The HUVEC cells that were treated with vehicle (control) served as negative controls. ^*^ *P*<0.05 vs. control group. ^**^ *P*<0.01 vs. control group. Values are expressed as mean±SEM. Similar results were obtained in three independent experiments.

DNA fragmentation and nucleosome depolymerization occurred during apoptosis in various cells. To construct a cell apoptosis model, HUVEC cells were treated with different concentrations of Ox-LDL and were analyzed by DNA fragmentation and cell proliferation assay. As shown in Fig. 4, Ox-LDL decreased cell viability and increased DNA fragmentation in a dose-dependent manner as analyzed in 24 h by Cell Proliferation and Cell Death Detection ELSIA kits. Furthermore, there was a significant difference in cell apoptosis when the concentration of Ox-LDL was as high as 100 µg/mL. In contrast, there was no obvious change in the cell viability and DNA fragmentation in controls. Therefore, the HUVEC cells treated with 100 µg/mL Ox-LDL for 24 h were used as cell apoptosis models in the rest of the studies.

### The Effects of the Silkworm Protein 30Kc6 on Viability and Apoptosis in HUVEC Cells Treated with Ox-LDL

**Figure 5:**
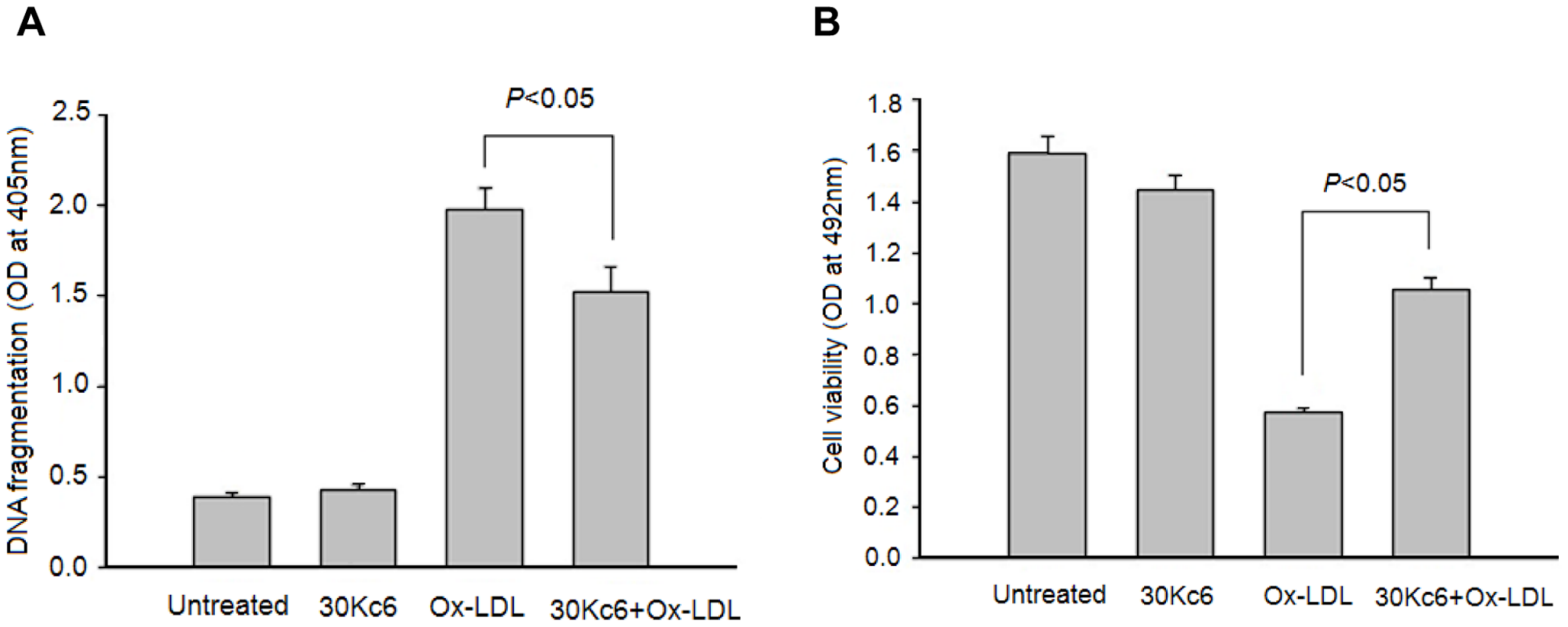
The effects of 30Kc6 on Ox-LDL-induced HUVEC cell apoptosis (A) and cell viability (B). “Untreated” represents the HUVEC group without treatment. The 30Kc6 represents the HUVEC group treated with 30Kc6. The Ox-LDL represents the HUVEC group treated with Ox-LDL. The 30Kc6+ Ox-LDL represents the HUVEC group treated with 30Kc6 and Ox-LDL. ^*^ *P*<0.05 compared with the indicated groups. Values are expressed as mean±SEM. Similar results were obtained in three independent experiments.

In order to explore the effects of the silkworm protein 30Kc6 on HUVEC cells, Ox-LDL-induced apoptosis was used in HUVEC cells in this study. The HUVEC cells were treated with Ox-LDL in the presence or absence of 30Kc6 and were then subjected to analysis of cell viability and DNA fragmentation using Cell Proliferation and Cell Death Detection ELISA kits. There was no obvious difference in cell viability and DNA fragmentation in the HUVEC cell treated with 30Kc6 and the ones without any treatment (Fig. 5). This was an indication that the 30Kc6 protein alone showed no obvious effect on cell growth or apoptosis. However, there were dramatic difference in cell viability and apoptosis between the vehicle control groups and the HUVEC cells treated with Ox-LDL. Interestingly, 30Kc6 dramatically inhibited Ox-LDL-induced cell apoptosis, while increased the cell viability in HUVEC cells. These data indicated that the silkworm protein 30Kc6 could inhibit cell apoptosis and enhanced cell viability in HUVEC cells, which were treated with Ox-LDL.

**Figure 6:**
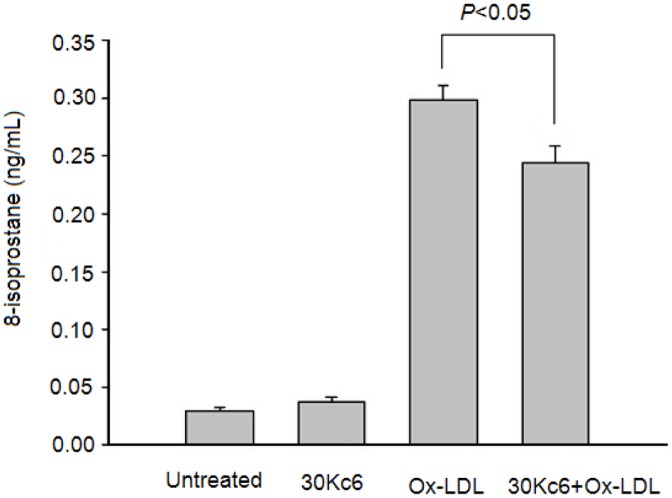
The effects of 30Kc6 on levels of 8-isoprostane in HUVEC cells incubated with Ox-LDL. The level of 8-isoprostane was analyzed in different groups. “Untreated” represents the HUVEC group without treatment. The 30Kc6 represents the HUVEC group treated with 30Kc6. The Ox-LDL represents the HUVEC group treated with Ox-LDL. The 30Kc6+ Ox-LDL represents the HUVEC group treated with 30Kc6 and Ox-LDL. ^*^ *P*<0.05 compared with the indicated groups. Values are expressed as mean±SEM. Similar results were obtained in three independent experiments.

The 8-isoprostan is a recognized marker of oxidative stress, which has a high level in cells undergoing oxidative stress and apoptosis [16]. To investigate whether the Ox-LDL can induce oxidative stress in HUVEC cells, the levels of 8-isoprostane were analyzed. As demonstrated in Fig. 6, Ox-LDL obviously increased the level of 8-isoprostane. However, there was no difference in the level of 8-isoprostane between the HUVEC cells without treatment and the cells treated with 30Kc6, indicating that 30Kc6 alone did not affect oxidative stress in this sutdy. In contrast, 30Kc6 significantly decreased the levels of 8-isoprostane and oxidative stress in HUVEC cells treated with Ox-LDL (*p*<0.01), suggesting that 30Kc6 might inhibit cell apoptosis by scavenging of reactive oxidative species and oxidative stress.

### The Effect of the Silkworm Protein 30Kc6 on the JNK and p38 MAP Kinase Activity

**Figure 7:**
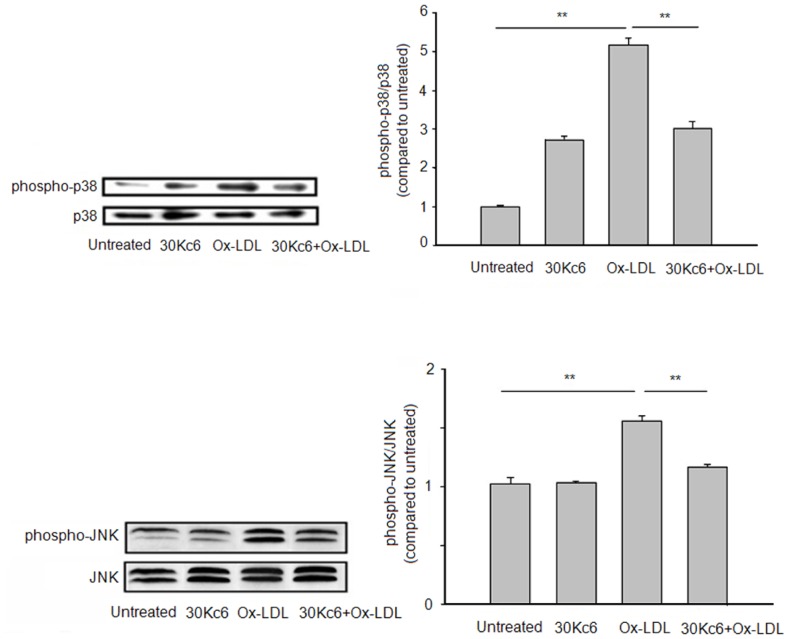
The effects of 30Kc6 on p38 (A) and JNK MAP kinase (B) activity after exposure to Ox-LDL. The HUVEC cells were treated and analyzed by Western blot. “Untreated” represents the HUVEC group without treatment. The 30Kc6 represents the HUVEC group treated with 30Kc6. The Ox-LDL represents the HUVEC group treated with Ox-LDL. The 30Kc6+ Ox-LDL represents the HUVEC group treated with 30Kc6 and Ox-LDL. The right panels show the results of densitometric analyses of immunoblotting (mean±SEM, n = 3). ^**^ *P*<0.01 compared with the indicated groups.

The MAP kinase family, especially the p38 and the JNK, have been reported to be involved in oxidative stress and cell apoptosis [10]–[12]. Therefore, the activities of p38 and JNK MAP kinase were examined by Western blot using antibodies (Fig. 7). The Ox-LDL significantly activated the p38 and the JNK MAP kinase in HUVEC cells that were incubated with Ox-LDL. Furthermore, the 30Kc6 dramatically decreased the level of the activated p38 and JNK induced by Ox-LDL (Fig. 7), indicating that p38 and JNK MAP kinase were involved in the effect of 30Kc6 on HUVEC apoptosis. 30Kc6 might prevent the Ox-LDL-induced cell apoptosis by decreasing activation of the p38 and the JNK MAP kinase in the HUVEC cells.

### Protein Concentration Analysis of 30Kc6 in Freeze-dried Silkworm Pupa

Protein concentration of 30Kc6 in freeze-dried silkworm pupa meals was analyzed by ELISA. To set up the standard curve between the concentration of purified 30Kc6 sample and the OD value, the 30Kc6 proteins were diluted by double dilution with the initial concentration of 5 µg/mL. The OD_492_ value was measured by ELISA and the standard curve was constructed with the concentration of the 30Kc6 protein as the horizontal ordinate and the OD_492_ value as the longitudinal coordinate. The linear-regression analysis demonstrated that the constructed standard curve complied with regression equation (y = 0.3401x+0.0282, R^2^ = 0.9987). According to the equation, the concentration of 30Kc6 was 9.71 mg/g.

### The Effects of 30Kc6 on Atherosclerotic Rabbit

The effect of the silkworm protein 30Kc6 was further examined *in vivo*. Atherosclerotic rabbit models were set up, treated with 30Kc6 protein and analyzed. The levels of serum TC, TG, HDL-C and LDL-C increased in the rabbits, which were fed with high fat diets in eight weeks, indicating that the experimental animals were in hyperlipidemia state. The aortas and livers in the randomly sacrificed rabbits showed some pathological changes.

After the following four weeks of different diet feeding, rabbit serum biochemical indicators of six diet groups with different treatments were assessed (Table 1). The levels of TC, TG, HDL-C and LDL-C were decreased in the groups treated with low and high dose of 30Kc6 as well as the positive controls. Specifically, the levels of TC, TG and LDL-C were significant different between the groups treated with low dose of 30Kc6 and Bacmid (*p*<0.05). Moreover, the levels of TC, TG and LDL-C (*p*<0.01) and HDL-C (*p*<0.05) were significantly decreased in the group treated with high dose of 30Kc6 as compared to the group treated with the Bacmid. This observation indicated that 30Kc6 had protective effects on decreasing the blood fat levels. The blood 30Kc6 concentration of rabbits was determined by ELISA after the oral administration of different diet at 2 h. There was no significant detectable amount in normal, Bacmid and positive groups. The average 30Kc6 serum concentration of high and low dose groups were 46.7 ng/L and 8.3 ng/L, respectively.

**Table 1:** Serum biochemical indicators in New Zealand white rabbits in 12 weeks (mg/dl).

Groups	TC	TG	LDL-C	HDL-C
Normal control group	44.89±7.3	57.57±18.60	28.87±3.56	22.43±3.89
High-fat group	710.53±45.27	135.52±15.94	677.22±74.52	70.77±7.35
Bacmid group	628.88±52.63	126.66±13.29	632.05±62.55	66.51±8.51
Low-dose group	533.67±17.80*	12.49±11.51*	562.16±54.44*	56.07±10.05
High-dose group	408.28±22.06^**^	94.77±19.49^**^	469.88±44.79^**^	52.59±8.12*
Positive control group	203.95±26.32^**^	69.09±14.17^**^	167.57±14.29^**^	30.55±3.96^**^

* mean significant difference at *p*<0.05 level, ^**^ mean significant difference at *p*<0.01 level.

**Figure 8:**
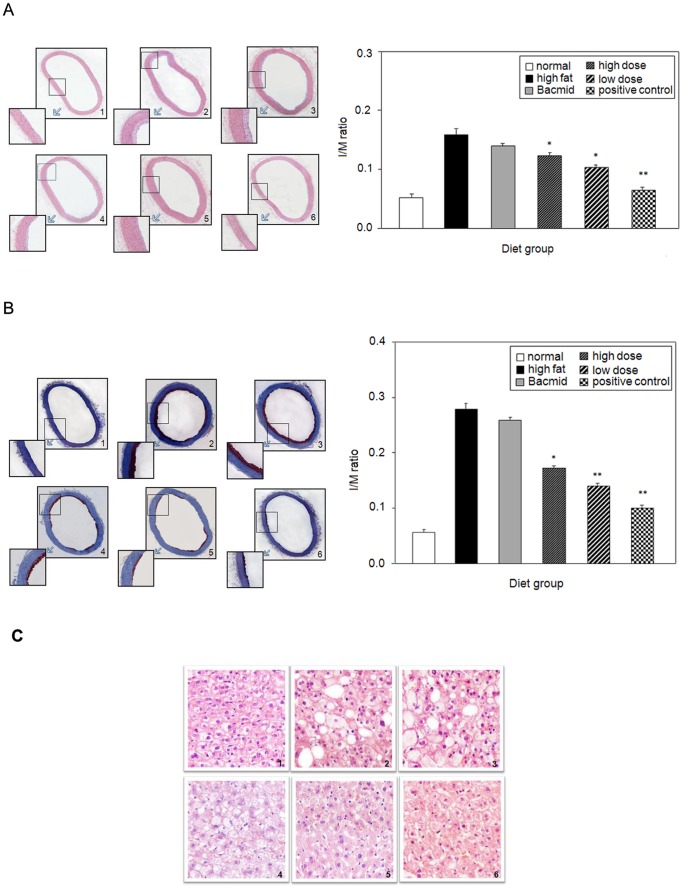
The effects of 30Kc6 on neointima formation of rabbit aortas. Atherosclerotic rabbits and controls were orally administered with different silkworm pupa meals and probucol, respectively for four weeks and the rabbit aortas were analyzed by HE (A) and red oil O staining (B). The histochemical analyses of livers were detected by HE staining (100×) (C). 1. Normal control group; 2. High-fat control group; 3. Bacmid group; 4. High-dose 30Kc6 group; 5. Low-dose 30Kc6 group; 6. Positive control group. Representative cross-sections with HE or oil red O staining (left panels) and morphometric analyses (right panels) are shown. ^*^ *P*<0.05 vs. High-fat control group and Bacmid group. ^**^ *P*<0.01 vs. High-fat control group and Bacmid group. Values are expressed as mean±SEM.

Then the atherosclerotic rabbits were sacrificed by gas embolism after drawing blood from their hearts. Aortas were isolated, stained with HE or oil red O and analyzed. Fig. 8A demonstrates that there were obvious atherosclerotic characteristics in the atherosclerosis rabbit groups fed with high-fat diet when analysed by HE stain. These characteristics included serious lesions in the intimas of the aortas and fused plaque areas that were spreading into intimas. In contrast, there was no atherosclerotic characteristic in the normal control group and the morphologies of aortas were normal with smooth intimas. However, compared to the rabbit groups treated with Bacmid, 30Kc6 significantly alleviated the atherosclerotic states of the atherosclerosis rabbits (Fig. 8A). Similar results were obtained when the samples were analyzed by red oil O staining (Fig. 8B).

As revealed in Fig. 8C, the liver structure and morphology were clear and normal in the normal control rabbit group. However, hepatic lesions were obvious in the rabbit groups which were fed with high fat diet. Furthermore, the vacuolization of liver cells was alleviated in the positive probucol-treated group and the groups that were fed with 30Kc6. These *in vivo* data further confirmed that 30Kc6 had protective effects on the atherosclerosis rabbits.

## Discussion

The 30K family proteins in silkworm Larva hemolymph are low density lipoproteins. The 30Kc6 has been confirmed to be a member of the 30K family proteins with an anti-apoptotic activity [3]. The 30K family proteins usually have both apolipoprotein and anti-apoptosis function [1]–[5]. Therefore, it is reasonable to deduce that the silkworm protein 30Kc6 might decrease the blood fats, dredge vessels, and protect blood vessel endothelium. It is necessary to investigate the effects of 30Kc6 on Ox-LDL-induced apoptosis in HUVEC cells.

The Ox-LDL led to oxidative stress-induced damage in HUVEC cells, which was regarded as an important step in the process of atherosclerosis. Therefore, prevention of the oxidative stress-induced damage in HUVEC cells is a major improvement in the prevention and treatment of atherosclerotic diseases [7], [9]. HUVEC is a direct target of Ox-LDL, so Ox-LDL-induced cell apoptosis in these cells was employed to simulate oxidative stress-induced damage in this study. DNA fragmentation is a typical characteristic of cell apoptosis [17]. Therefore, the effects of 30Kc6 on Ox-LDL-induced cell viability and intracellular DNA fragmentation were explored in order to analyze the protective roles of the 30Kc6 protein. Our data demonstrated that the silkworm protein 30Kc6 prevented Ox-LDL-induced damage and apoptosis in HUVEC cells by decreasing the oxidative stress and inhibiting the activation of p38 MAP and JNK MAP kinases.

The most striking question in producing proteins and peptides by silkworm bioreactor has been the oral administration of these products in recent years. Various protein and peptide drugs were used in the form of injections. However, the oral delivery of protein drugs and vaccines produced in silkworm pupa by genetic engineering has been most successful in clinical experiments and animal tests [18]–[20]. It is believed that only the peptides absorbed by intestinal tracts play physiological roles in traditional theory. Unfortunately, most proteins could not be absorbed by intestinal tracts and thus could not play role under various physiological and pathological conditions. Therefore, oral administration is important both in theory and application.

In this study, atherosclerotic rabbit models were constructed and fed with silkworm pupa containing 30Kc6 proteins. Serum proteins, aortas and liver tissues were all measured in the atherosclerotic rabbit models. Our data showed that the Bacmid-infected silkworm pupa contained a certain amount of natural 30K protein. When compared to the high-fat group, the serum biochemical indicators of rabbit model decreased to some extent after oral administration, but did not result in a statistically significant difference. However, compared with the high-fat group, the blood biochemical parameters were significantly different in case of oral administration of 30Kc6 freeze-dried silkworm pupa powder in a rabbit model. In conclusion, our results showed that oral administration of 30Kc6 silkworm pupa had certain preventive and therapeutic effects on atherosclerotic rabbit models, providing meaningful information for the prevention and treatment of atherosclerosis in clinical application.
